# Efficacy of COVID-19 Vaccination in People Living with HIV: A Public Health Fundamental Tool for the Protection of Patients and the Correct Management of Infection

**DOI:** 10.3390/idr14050080

**Published:** 2022-10-17

**Authors:** Alessio Facciolà, Smeralda D’Amato, Sebastiano Calimeri, Daniela Lo Giudice, Cristina Micali, Ylenia Russotto, Emmanuele Venanzi Rullo, Giuseppe Nunnari, Raffaele Squeri, Giovanni Francesco Pellicanò

**Affiliations:** 1 Department of Biomedical and Dental Sciences and Morphofunctional Imaging, University of Messina, 98124 Messina, Italy; 2 Department of Clinical and Experimental Medicine, University of Messina, 98124 Messina, Italy; 3 Department of Human Pathology of the Adult and the Developmental Age “G. Barresi”, University of Messina, 98124 Messina, Italy

**Keywords:** COVID-19, HIV/AIDS, vaccination, efficacy

## Abstract

HIV/AIDS is considered a risk factor for increased mortality due to COVID-19. For this reason, it is essential to include this population in vaccination campaigns. Studies found that antibodies are lower in HIV+ patients than in healthy individuals. The aim of this study was to assess the immune response in a cohort of people living with HIV/AIDS (PLWH) vaccinated with COVID-19 vaccination in order to evaluate the role played by the HIV infection in the efficacy of this vaccine. We carried out a cross-sectional study in the period April-September 2021, involving a cohort of PLWH and a cohort of HIV-uninfected people as the control group. The efficacy of vaccination was high in both groups despite a slight and not significant difference between them. However, important differences were found according to the intensity of the immune response. Specifically, while in the HIV+ group almost a quarter of people had a low response, it is important to remark that the control group had only a high or intermediate response after vaccination. Our results suggest the high efficacy of the mRNA COVID-19 vaccine in PLWH and the importance to vaccinate against COVID-19 in these patients in order to increase their protection.

## 1. Introduction

The coronavirus disease 2019 (COVID-19) has rapidly spread worldwide with a number of different variants that, according to the World Health Organization (WHO), have caused 616,427,419 confirmed cases and 6,528,557 deaths until now (last data of 5 October 2022) [1,2]. Since its onset, SARS-CoV-2 showed high transmissibility and high mortality rates, which have mostly been observed in vulnerable people such as the elderly, people with comorbidities such as diabetes, cardiovascular diseases, and obesity and immunosuppressed patients but also in pregnant women [3,4,5].

Thanks to Highly Active Antiretroviral Therapy (HAART), HIV infection is now a manageable disease turned into a chronic clinical condition with high life expectancy and a mortality rate dramatically reduced compared to the past. However, the increase in life expectancy but also the HAART itself due to its side effects, has led over time to an increase in the incidence of some comorbidities such as diabetes, cardiovascular diseases, kidney diseases and malignancies among people living with HIV (PLWH) [6,7,8,9,10,11,12,13,14,15]. Therefore, PLWH, because of the high incidence of chronic diseases, are at high risk of COVID-19 infection and serious clinical outcomes. Information about the epidemiology and trend of COVID-19 in PLWH is currently evolving [16]. However, HIV/AIDS is to be considered an independent risk factor for increased mortality due to COVID-19 [17]. PLWH have been disproportionately affected by COVID-19 and are at increased risk for severe clinical symptoms and mortality due to SARS-CoV-2 infection, especially among those with lower CD4+ T cell counts or detectable HIV viremia [18,19,20,21]. For this reason, it is crucial to include this risk population in the vaccination campaigns against COVID-19.

With the emergence of COVID-19 vaccines, vulnerable populations, including PLWH, require particular attention and should be prioritized for vaccination to reduce the number of infections and deaths in this population. Vaccination is essential to face the COVID-19 pandemic [22]. In Italy, access to COVID-19 vaccination has been extended to the population at risk for severe outcomes, including PLWH, starting from March 2021, using the BNT162b2 COVID-19 mRNA vaccine (Pfizer/Biontech) [23]. However, most COVID-19 vaccine studies did not include immunocompromised individuals, so there are few available data on vaccine efficacy in PLWH [24]. There is wide evidence that immunodeficient individuals are likely to have a suboptimal vaccine response and less lasting protection than the general population [25,26]. Concerning COVID-19 vaccination in PLWH, some data have been recently published. Overall, the initial experience appears to be positive in terms of safety and immunogenicity in a limited follow-up [27,28]. According to a recently published prospective study including 143 HIV patients aged above 18 years and 261 healthy Health Care Workers (HCWs) used as control, it was found that 18 days after the second dose of BNT162b2 vaccination, 98% of HIV patients had positive anti-receptor binding domain (RBD) IgG while 98.9% HCWs had positive anti-RBD IgG 26 days after the second dose of BNT162b2 vaccine [27]. The efficacy of vaccinations in such patients is largely related to CD4+ cell count, viral load, and disease stage [27,29]. It is also found that spike levels of IgG antibodies are lower in HIV+ patients than in healthy individuals [30].

The aim of this study was to assess the immune response in a cohort of PLWH vaccinated with the primary cycle of COVID-19 vaccination in order to evaluate the potential role played by the HIV infection in the efficacy of this vaccine.

## 2. Materials and Methods

The study was conducted at the University Hospital “G. Martino” of Messina, Italy, with the collaboration of the Unit of Infectious Diseases, where all the enrolled PLWH are followed, and the Operative Unit of Hospital Hygiene where vaccinations were carried out. We carried out a cross-sectional study from April to September 2021, involving a cohort of PLWH and a cohort of HIV-uninfected HCWs as a control group. The participants from both groups had never contracted COVID-19 (condition evaluated through medical history and preliminary search of anti-nucleocapsid antibodies, markers of natural infection) and all were subjected to the primary cycle of COVID-19 vaccination consisting of two doses of BNT162b2 COVID-19 mRNA (Pfizer/Biontech), NIAID’s mRNA-1273 (Moderna) and ChAdOx1-nCoV-19 (AstraZeneca) vaccines. The control group, perfectly matched for age and sex with the sample, was composed of HIV-uninfected HCWs randomly chosen among the ones vaccinated by our Operative Unit. Antibodies against Spike protein were detected one month after the completion of the primary cycle of vaccination. To this aim, after obtaining informed consent to participate in the study, blood samples were collected and centrifuged at 4000 rpm for 10 min and a CLIA (ChemiLuminescence ImmunoAssay) test (LIAISON SARS-CoV-2 S1/S2 IgG—DIASORIN S.p.A., Saluggia, Italia) consisting in a quantitative assay for the detection of IgG antibodies against S1/S2 antigens of SARS-CoV-2 was used. Specifically, values <33.7 AU/mL are considered negative. The maximum value of the test was >2080 AU/mL.

### Statistical Analyses

All the obtained data were collected and analyzed with Prism 4.0 software (Macintosh, San Diego, CA, USA). Descriptive statistics were used to find percentages, mean values and standard deviations. The comparisons between the groups under study were carried out through correlation tests, a chi-square test and Student’s *t*-test. Significance was assessed at the *p* < 0.05 level.

## 3. Results

The sample of PLWH enrolled in this study was composed of 84 people, all of them on HAART, with 85.1% men. The mean age of the sample was 48 ± 13.2 (min. 21; max. 74). The control group was composed of 64 HIV-uninfected HCWs, all of Italian nationality, of which 80.5% were men. The mean age was 42.1% ± 12.1 (min. 27; max. 67). The detailed composition of the two samples is shown in Table 1.

In the PLWH group, the majority of the participants were of Italian origin; only 9.6% were foreigners coming from Africa (50%) and Eastern Europe (50%). However, we have to highlight that while the Italian group was composed almost exclusively of men, in the foreigners the majority of the sample (75.0%) was represented by women. Foreigners women represented 60% of all the women in the HIV+ group. Moreover, remarkable mean age differences were present within the group between men and women. In general, women were ten years younger than men and foreigners were almost fifteen years younger than Italians. These gaps were most accentuated when comparing Italian and foreign men with an age difference of about eighteen years between these two groups while this difference was less evident between Italian and foreign women (only six years). The mean age of infection duration was 12 years.

According to the laboratory parameters, 86.9% of the whole HIV+ sample had no circulating viral load (TND) while the mean values of white cells, CD4+, CD4+/CD8+ ratio and CD34+ were, respectively 6942 ± 2235 (min. 700; max. 12,500), 832 ± 375 (min. 10; max. 1917), 1.12 ± 0.53 (min. 0.02; max. 2.56), 2.34 ± 1.83 (min. 0.00; max. 7.03). The detailed laboratory parameters of the HIV+ sample according to gender and nationality are shown in Table 2.

Table 2 shows that all the laboratory parameters reflected a well-controlled infection. The majority of the sample had no circulating viral load with very similar percentage values between Italians and foreigners and between men and women. In a high percentage (95%), those with a detectable circulating viral load had a value of <1000 copies/mL. Only one case was in the AIDS phase with a circulating viral load of 2,000,000 copies/mL, a CD4+ count of 10/mm^3^, and a CD4+/CD8+ ratio of 0.02.

In the HIV+ group, 45.2% of people stated to suffer from comorbidities, of which 44.7% were affected by dyslipidemia, 34.2% by hypertension, 23.7% by diabetes (the three clinical conditions were present at the same time in different combinations in 31.6% of the patients). Only a very small percentage of patients was affected by immune disorders such as autoimmune diseases (10.5%) or clinical conditions negatively affecting the immune system such as malignancies (7.9%). In the control group, almost one-third (29.3%) stated that were affected by comorbidities such as hypertension (45.6%), dyslipidemia (12.8%) and diabetes (21.7%).

**Figure 1:**
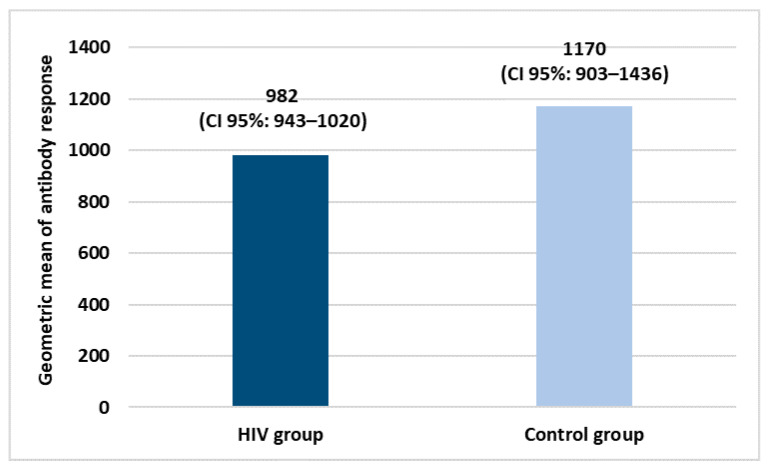
Geometric means of antibody response after COVID-19 vaccination primary cycle.

Both groups received two doses of COVID-19 vaccination. Specifically, in the HIV+ group, 84.3% of subjects were vaccinated with BNT162b2 COVID-19 mRNA (Pfizer/Biontech), 8.4% with ChAdOx1-nCoV-19 (AstraZeneca) and 3.6% with NIAID’s mRNA-1273 (Moderna). All the subjects belonging to the control group were vaccinated with BNT162b2 COVID-19 mRNA (Pfizer/Biontech). Figure 1 shows the geometric mean of the antibody response in both study groups.

Evaluating the geometric means of antibody response, vaccination has good efficacy in both groups with a slight and not significant difference between them.

**Figure 2:**
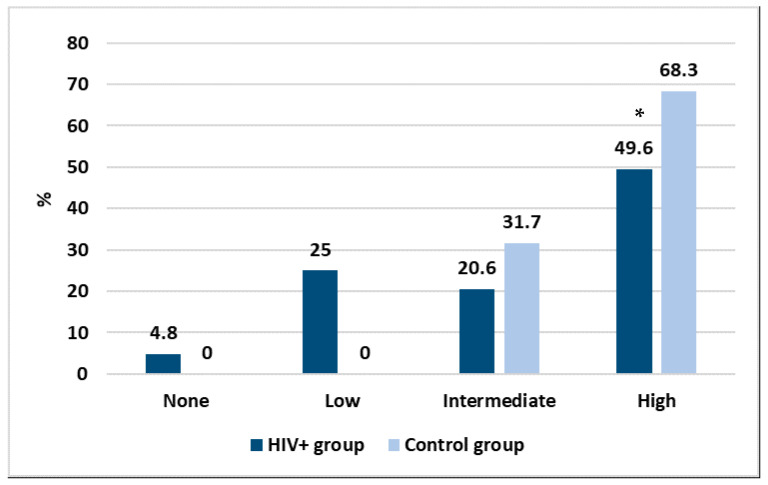
Intensity in the antibody response between the two studied groups, * *p* < 0.05.

With the purpose to create a discriminating method allowing us to better express the results and to make them more comparable, we grouped the antibody response as high (≥2080 AU/mL), intermediate (500–2079 AU/mL), low (33–499 AU/mL) and none (<33 AU/mL). The results are shown in Figure 2.

Considering those who did not have any antibody response, Figure 2 shows that vaccination had an efficacy of 95.2% in the HIV+ group and 100% in the control group. However, important differences were found according to the intensity of the immune response. Specifically, while in the HIV+ group almost a quarter of people had a low response, in the control group all had a high or intermediate response after vaccination. Moreover, a statistically significant difference was found between the two groups in the high response (*p* < 0.01). No correlation was found between the intensity of the immune response and personal data or any of the laboratory parameters.

**Figure 3:**
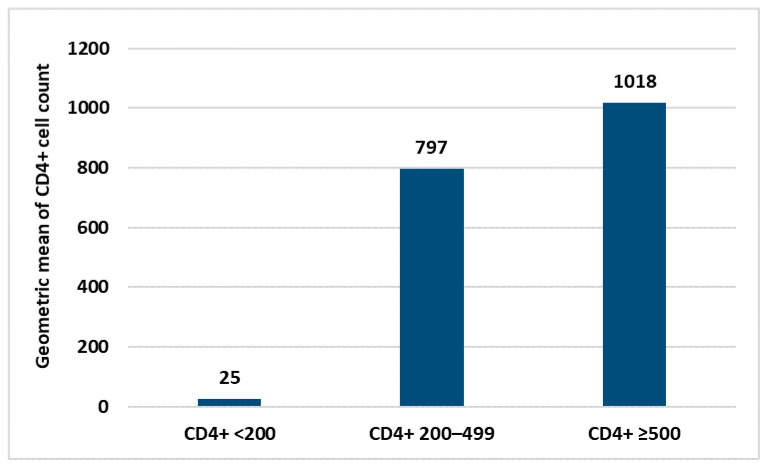
Difference in the immune response according to the mean values of CD4+ count.

Within the HIV+ group, no statistical correlation was found between antibody response and personal data (age, sex and nationality) neither with laboratory parameters nor the type of COVID-19 vaccine used. The only laboratory parameter correlated with the intensity of the immune response was the CD4+ count (Figure 3). Similarly, in the control group, no correlation between personal data and immune response was found.

## 4. Discussion

Despite differences among published studies, PLWH seems to be a high-risk group for COVID-19 negative clinical outcomes and higher hospitalization and mortality rates [31,32,33,34]. This issue could be the consequence of both poor immune responses to SARS-CoV-2 natural infection [35] and to the contemporary presence of higher rates of chronic comorbidities and low socioeconomic status [36]. These findings joined with the observation that HIV infection could negatively affect the serological response to other viral agents, such as influenza [37], lead us to evaluate the effectiveness of the COVID-19 vaccine in this group of people. The lack of information about the effectiveness of the COVID-19 vaccine among PLWH is partially due to the exclusion of these patients in the initial vaccine effectiveness trials [38].

Some studies focused on both the efficacy of prime–boost dosing of the ChAdOx1-nCoV-19 (AstraZeneca) vaccine in PLWH on HAART treatment with CD4+ counts >350 cells/mm^3^ [39] and its persistence after 6 months from vaccination [40]. These studies showed a decrease both in humoral and cellular immunity at 6 months, despite no significant difference being found compared to a cohort of HIV-uninfected people vaccinated with the same vaccine. To date, only few observational studies were carried out about the effectiveness of mRNA vaccines in this group of patients, and all showed a good humoral and cellular immune response after the primary vaccination cycle in patients on HAART and with CD4+ cell counts >200 cell/mm^3^ [41,42,43]. However, the response is significantly lower in PLWH with CD4+ cell counts <200/mm^3^ compared to those with a count >500 cell/mm^3^ and HIV-uninfected controls. These results suggest that reduced CD4 counts correlate with reduced concentrations of antibodies to SARS-CoV-2 [44]. Therefore, CD4+ counts, uncontrolled viral load and disease severity can affect the efficacy of vaccination [45,46,47].

Like other geographical settings, HIV infection in our territory showed a change in the incidence with an increase in cases in heterosexuals and in more advanced age due to late diagnosis in this group of people [48]. Moreover, the efficacy of HAART has extended the life expectancy of these patients with the possible onset of many different comorbidities. To date, HIV+ people with a long story of HIV infection could be at high risk to get COVID-19 and have adverse events, and for this reason, it is crucial to consider this group of patients in the COVID-19 vaccination campaigns.

In our study, a group of HIV+ patients were vaccinated against COVID-19 and studied comparing them with a control group of HIV-uninfected people represented by a cohort of HCWs. HCWs are at the same time a category at high risk to get COVID-19 and are widely studied from the point of view of the management of SARS-CoV-2 infection and immune response after vaccination [49,50]. The results showed a general good efficacy of the used vaccines in both groups. However, we found a remarkable difference between the two groups regarding the intensity of the immune response. Indeed, while the control group had only high and intermediate immune responses to the vaccination, in the HIV+ group we had a more heterogeneous response with almost one-quarter of people that showed a low response. This response was not correlated with personal data (age, sex, presence of comorbidities) in either of the two groups. Considering the very similar composition of the control group regarding personal data and the presence of comorbidities, this observed difference in the elicitation of immunity by the vaccination could be explained by the HIV infection. Many previous studies have highlighted an HIV-infected T-cell dysfunction that cannot be fully restored despite the benefits of HAART. For example, during chronic HIV infection, T-cell exhaustion characterized by progressive loss of cell proliferation and effector functions, metabolic dysregulation, increased inhibitory receptor expression, and distinct transcriptional signatures occur due to chronic exposure to viral antigens, inflammatory signals, and/or cell-intrinsic defects [51,52,53,54,55]. Metabolic dysregulation occurring during chronic HIV infection includes declines in glucose uptake, progressive mitochondrial damage, and increased reactive oxygen species (ROS) production. All these modifications probably contribute to accelerating T-cell aging, senescence, and apoptosis [56,57,58,59].

According to our findings, in order to offer complete protection to this group of potentially vulnerable populations, we can state that COVID-19 vaccination must be considered an essential part of all those preventive tools represented by the administration of other vaccinations (i.e., HPV, HBV, influenza, pneumococcal and meningococcal vaccines) [60], the increase in cancer screening tests [61,62], and finally the improvement of the knowledge about sexually transmitted infection [63], as well as the development of new and more and more effective drugs [64].

## 5. Conclusions

Our results suggest the very high efficacy of mRNA COVID-19 vaccines in HIV-infected people on HAART and with a well-controlled disease (undetectable viremia and CD4+ count >500/mm^3^). This suggests the importance to vaccinate PLWH against COVID-19 to which these patients seem to be more susceptible in terms of morbidity and mortality compared to HIV-uninfected people. These findings are very important also in light of the very fast mutation rate of SARS-CoV-2, with the emergence of different variants of concern (VOC) that can be a great threat to all vulnerable categories of populations. The fight against the COVID-19 pandemic is still ongoing and the recent introduction of vaccines against the last variants can represent a further weapon in this unprecedented battle. Therefore, further booster doses with these new vaccines are strictly recommended in this vulnerable population, which helps us to more effectively protect PLWH.

## Figures and Tables

**Table 1:** Composition of the samples according to mean age, gender and nationality.

**PLWH (*n* = 84)**		
**Whole sample**	%	Mean age
*Men*	85.1	49.2 ± 12.4 (min. 25; max. 74)
*Women*	14.9	39.1 ± 14.7 (min. 21; max. 67)
**Nationality**		
*Italians*	90.4	49.3 ± 12.3 (min. 25; max. 74)
*Foreigners*	9.6	35.5 ± 14.2 (min. 21; max. 67)
**Gender/Nationality**		
*Italian men*	94.7	49.7 ± 12.3 (min. 25; max. 74)
*Italian women*	5.3	42.8 ± 11.1 (min. 28; max. 57)
*Foreign men*	25.0	32.0 ± 1.6 (min. 30; max. 34)
*Foreign women*	75.0	36.7 ± 16.2 (min. 21; max. 67)
**CONTROL GROUP (*n* = 64)**		
**Whole sample**	%	Mean age
*Men*	80.5	43.3 ± 12.2 (min. 27; max. 67)
*Women*	19.5	37.0 ± 10.1 (min. 29; max. 58)

**Table 2:** Details of laboratory parameters in the HIV+ sample according to gender and nationality.

	**TND (%)**	**WBC (Mean Value in Absolute Counts/mm^3^ ± SD)**	**CD4+ (Mean Value in Absolute Counts/mm^3^ ± SD)**	**CD4+/CD8+ Ratio (Mean Value in Absolute Counts/mm^3^ ± SD)**
**Nationality**				
*Italians*	86.8	7002 ± 2262 (min. 700; max. 12,500)	827 ± 387 (min. 10; max. 1917)	1.08 ± 0.5 (min. 0.02; max. 2.36)
*Foreigners*	87.5	6371 ± 1897 (min. 3600; max. 9970)	878 ± 223 (min. 492; max. 1069)	1.55 ± 0.6 (min. 0.73; max. 2.56)
**Gender/Nationality**				
*Italian men*	86.1	6936 ± 2208 (min. 700; max. 12,500)	814 ± 386 (min. 10; max. 1917)	1.07 ± 0.5 (min. 0.02; max. 2.36)
*Italian women*	75.0	8183 ± 2812 (min. 3700; max. 10,600)	1055 ± 345 (min. 541; max. 1482)	1.18 ± 0.64 (min. 0.25; max. 2.05)
*Foreign men*	100.0	8785 ± 1185 (min. 7600; max. 9970)	1025 ± 44 (min. 980; max. 1069)	1.74 ± 0.11 (min. 1.63; max. 1.85)
*Foreign women*	83.3	5567 ± 1261 (min. 3600; max. 7200)	830 ± 237 (min. 492; max. 1056)	1.49 ± 0.67 (min. 0.73; max. 2.56)

## Data Availability

Not applicable.
